# A Wife Worth Having

**Published:** 1876-04

**Authors:** 


### A WIFE WORTH HAVING.

One of our lady correspondents writes: "The June number of the Journal of Health is filled with articles unusually interesting. '*The Watchful Mother*,' has strengthened my former resolutions. At present, I do all my own work, cook for five in family, sweep, dust, and build fires; take care of my two little ones, teach eight piano pupils, giving to each two hours a week, give three lessons a week to a class in vocal music, besides classes in the school room, several hours every day. In addition, I canvass for pupils, receive our friends, retire at half past eleven, and rise at five in the morning. But I find my eyes growing heavy, and my bones ache with servitude."

Who does not feel that a woman of such energy ought to succeed? Who does not regret that she should be called to perform labors so multifarious and so incongruous. In view of this, there are multitudes of married women not "wives," who may well hide their faces in shame, who, with no larger family, have a cook and housemaid, and yet, are ceaselessly complaining of how much trouble they have, how they are worn out with work; who can dilate indefinitely on the hardness of their lot, and who, without earning a dollar a week, complain of being tired of living in such destitution, and cry and pout by the hour, whenever a coveted silk dress, or beauty of a bonnet, or love of a point lace collar or cuff is not procured, on the slightest intimation of being wanted—we do not say "asked for." There are women who think themselves descending, to ask their husbands for anything; who want money placed where they can get it at will, without any account of its expenditure; women, who in the vacillation of business, meet the prudent suggestions of retrenchment with impatient reproaches, if not with downright epithet and rage; who never inquire, "Can we afford it?" who cannot brook the delay of a few days, until the "quarter's rent" is paid; who would not fail to be present at the "opening" of an autocratic milliner, even if it risked their husband a bank protest.

What was the manner of the rearing of such wives? As daughters, they were allowed to have their own way; every wish was gratified, every obstacle was removed from their path without any effort of their own. They were never allowed the opportunity of a self denial, and were practically taught that the convenience and comfort of mother, father, brothers, every body, must be sacrificed to their own; hence they grew up selfish, impatient of control, and, too often, to their own undoing and that of their husbands.

